# An Evaluation of Factors Predicting Diet Quality among Cancer Patients

**DOI:** 10.3390/nu10081019

**Published:** 2018-08-04

**Authors:** Kathleen Kane, Sanja Ilic, Holly Paden, Maryam Lustberg, Cassandra Grenade, Aashish Bhatt, Dayssy Diaz, Anna Beery, Irene Hatsu

**Affiliations:** 1 Department of Human Sciences, Ohio State University, Columbus, OH 43210, USA; kane.307@osu.edu (K.K.); ilic.2@osu.edu (S.I.); paden.10@osu.edu (H.P.); 2 Department of Internal Medicine, College of Medicine, Ohio State University, Columbus, OH 43210, USA; maryam.lustberg@osumc.edu (M.L.); cassandra.grenade@osumc.edu (C.G.); 3 Department of Radiation Oncology, College of Medicine, Ohio State University, Columbus, OH 43210, USA; aashish.bhatt@osumc.edu (A.B.); dayssy.diazpardo@osumc.edu (D.D.); anna.beery@osumc.edu (A.B.)

**Keywords:** cancer, diet quality, healthy eating index

## Abstract

A high diet quality is associated with a lower risk of cancer mortality. However, the predictive factors of diet quality among cancer patients are not well understood. This study determines the socio-demographic and disease-related factors that affect diet quality among cancer patients. Two hundred and forty-two cancer patients completed questionnaires assessing sociodemographic and disease-related characteristics. Diet quality was measured using the Healthy Eating Index 2010 (HEI). Independent sample *t*-tests and one-way ANOVA with post-hoc analysis using the Tukey HSD test were used to compare mean HEI scores across these characteristics. A regression model was used to determine factors that predicted diet quality. The overall HEI score among cancer patients was 61.59 (SD = 11.67). Patients with a high school degree or General Education Diploma (GED) or less had lower HEI scores (*β* = −4.03, *p* = 0.04; *β* = −7.77, *p* = 0.001, respectively) compared to those with college degrees. Additionally, homemakers had significantly higher HEI scores (*β* = 7.95, *p* = 0.008) compared to those who worked at least 40 hours per week. Also, individuals with some types of cancers (e.g., endometrial or uterine) had significantly higher HEI scores (*β* = 12.56, *p* = 0.002) than those with other cancers (e.g., head and neck). Our findings will help oncology healthcare providers identify and target cancer patients with specific demographic characteristics who are at increased risk for consuming poor-quality diets with much needed food resource interventions.

## 1. Introduction

Dietary factors account for the onset of several chronic diseases including cancer [1]. The consumption of poor diets also affects the progression, management, and outcome of these diseases [1]. The assessment of dietary consumption patterns can be achieved by the determination of individual food and nutrient intake. However, because of the synergistic way various dietary components function in the body, an evaluation of the overall diet based on a summary index may provide a more comprehensive analysis [2]. Diet quality is a concept used in evaluating and summarizing a population’s dietary habits and patterns in relation to factors such as compliance to dietary guidelines and nutrient adequacy. The Healthy Eating Index (HEI) is a validated multi-dimensional index of diet quality based on adherence to the Dietary Guidelines for Americans (DGA). Several studies have shown a higher HEI to be associated with a lower BMI, higher physical activity levels, a decrease in obesity prevalence, and a lower likelihood of smoking [3].

There is evidence of an association between a high diet quality and a decreased risk of chronic disease, including cardiovascular disease and cancer [4,5]. Among cancer patients, several studies show a high diet quality to be associated with a lower risk of cancer mortality [6,7,8,9], although there is some evidence that contradicts these findings [10,11]. Assessing diet quality among patients with chronic diseases is crucial for monitoring dietary changes in order to develop relevant nutrition interventions to improve disease outcomes [12]. Of equal importance to these interventions is an understanding of factors that may affect diet quality. Many studies have evaluated the predictive factors of diet quality in a multitude of population groups [8,13,14,15,16]. Cancer patients may differ clinically and demographically from other population groups; however, the predictive effect of these characteristics on a higher diet quality within this population have not been well elucidated. This study aims to determine the socio-demographic as well as disease-related predictors of diet quality among cancer patients. The study’s findings may help healthcare providers to identify patients at risk for consuming low-quality diets, which are, therefore, in need of targeted interventions that may help improve their diet quality.

## 2. Materials and Methods

### 2.1. Study Design and Sample

This observational study was conducted according to the Declaration of Helsinki and was approved by the Ohio State University Institutional Review Board (Project identification code: 2016C0013). It included a consecutive convenient sample of cancer patients diagnosed with any stage (I–IV) of the disease. Participants were recruited from three cancer clinics in central Ohio, USA from June 2016 to August 2017. Recruitment methods included flyers, referrals, and word of mouth. To be eligible for the study, participants had to be: (1) 18 years old or older, (2) diagnosed with cancer (Stages I–IV), (3) currently receiving treatment (surgery, radiation, all systemic therapies, etc.), (4) at any stage of treatment/therapy, and (5) willing and able to give a written informed consent. The only exclusion criterion was the refusal or inability to provide informed consent.

Potential participants were approached at the cancer clinics by research assistants and presented with a full description of the research protocol. Individuals who agreed to study participation were included in the study. After providing consent, each participant completed the study surveys, which included sociodemographic questionnaires, disease and treatment characteristics questionnaires, and a food frequency questionnaire. All questionnaires used in data collection were self-administered; however, trained research assistants provided assistance when needed. Participants received a $15 gift card for a local grocery store as an incentive to participation.

### 2.2. Measures

#### 2.2.1. Socio-Demographic Characteristics

Socio-demographic characteristics were assessed using surveys that evaluated data pertaining to gender, age, ethnicity, education, income, household dynamics, health insurance, use of food assistance programs, smoking status, drug use, and alcohol consumption. To assess the extent of financial burden that cancer poses on patients, timeliness of bill payment was included in the sociodemographic characteristics. The presence of financial strain can impact the amount of money allotted for food, with implication for diet quality. Questions were presented in multiple-choice and open-ended text formats.

#### 2.2.2. Disease and Treatment Characteristics

A battery of questions was used to evaluate the type and stage of cancer, as well as the time since diagnosis.

#### 2.2.3. Dietary Assessment

The usual amounts of nutrients and food groups consumed by each participant were estimated using the full-length six-month Block Food Frequency Questionnaire (FFQ). The Block FFQ is a validated questionnaire that assesses dietary and nutrient intake over a six-month period based on approximately 127 food items [17]. It also has additional questions that are used to adjust for fat, protein, carbohydrate, sugar, and whole grain content.

The information about nutrients and food intake was used in calculating Healthy Eating Index 2010 (HEI) scores. The HEI is a validated indicator of diet quality and reflects adherence to the federal Dietary Guidelines for Americans (DGA) [18]. It was used in this study, instead of a cancer-specific index, to allow for the evaluation of participants’ dietary patterns based on DGA recommendations and also to be able to compare their diet quality to that of the general adult US population. The HEI score is calculated from 12 component scores: nine components monitor foods and nutrients to be consumed in adequate amounts and three components monitor those to be consumed in moderation. The adequacy and moderation standards are based on the DGA 2010 and are described elsewhere [18]. Components of the adequacy category include: total fruit, whole fruit, total vegetables, greens and beans, seafood and plant proteins, and total protein foods, all of which have a score range of 0–5, as well as whole grains, dairy, and fatty acids which have a score range of 0–10. Components of the moderation category are: refined grains (0–10 points), sodium (0–10 points), and empty calories (0–20 points) [18]. A higher score in the adequacy components indicates higher consumption, while a higher score in the moderation category corresponds to lower consumption [19]. The overall HEI score ranges from 0 to 100, and diet quality improves as the HEI score increases. The scoring method for HEI-2010 is described elsewhere [20].

### 2.3. Statistical Analysis

Sample size was calculated on the basis of a medium effect size (0.15) [21]. A total sample of 230 participants was needed to detect medium effects at a power of 0.95 with an alpha at 0.05. On the basis of our previous experiences with the study population, we expected approximately 10–15% of our patients to leave the questionnaires incomplete.

A priori sociodemographic, health-related behavior characteristics, as well as disease and treatment-related characteristics that could potentially predict diet quality were selected and assessed according to the literature [13,14,15]. Independent sample t-tests and one-way ANOVA and post-hoc analysis using the Tukey HSD test were used to compare mean HEI scores across these characteristics. The variables that were significantly associated with diet quality in the unadjusted analysis were selected for inclusion in a multiple linear regression model. The dependent variable in the model was the HEI score. The results from the multiple regression model are summarized as coefficients, standard errors, and associated *p*-values. Statistical analyses were performed using SPSS version 24 (IBM Corp, Armonk, NY, USA).

## 3. Results

The study included 242 individuals diagnosed with cancer, two-thirds of whom were female. Approximately 87% of the sample identified as white non-Hispanic, with 10% being Black or African-American. The majority (60%) of participants were married, while 27% were divorced or widowed. About half of the sample (50.6%) had private insurance, while the remaining half had some form of public insurance (i.e., Medicare or public non-Medicare), and about 1% had no insurance. In addition, nearly 25% of participants paid their bills late because of medical expenses; less than 10% of participants had to borrow money to pay for medical expenses. A detailed description of the demographic and lifestyle behavior characteristics of the study sample is shown in Table 1. Table 2 describes the participants’ diagnosis- and treatment-related characteristics. Both tables also show the differences in diet quality among participants by these characteristics.

The average HEI score for the study sample was 61.59 (SD = 11.67, range: 33.93–90.72), which is significantly higher than that reported for the general U.S. adult population, (mean: 49.60, 95% CI: 48.9–50.4), based on the 2009–2010 wave of National Health and Nutrition Examination Survey (NHANES) data [22]. A comparison of component scores also showed a similar trend in all areas except for the fatty acid and sodium component scores, for which the study cohort’s averages were lower and hence worse than those of the general adult population (Table 3) [22]. These findings are not surprising, since individuals diagnosed with cancer tend to improve their dietary habits [23].

In bivariate analysis, gender (*p* = 0.006), age (*p* = 0.036), marital status (*p* = 0.007), level of education (*p* < 0.0005), employment (*p* = 0.002), monthly household income (*p* = 0.001), paying bills late due to medical expenses (*p* = 0.001), use of federal food assistance programs (*p* = 0.004), having private food assistance resources (*p* = 0.001), smoking status (*p* < 0.0005), and type of cancer (*p* < 0.0005) were significantly associated with diet quality (Table 1 and Table 2). A post-hoc comparison using the Tukey HSD test showed a significant difference in diet quality scores between individuals who were married (M = 63.63, SD = 11.96) compared to those who were divorced or widowed (M = 58.13, SD = 11.04). Those with less than a high school degree (M = 58.58, SD =10.63) and those with a high school degree or GED (M = 56.43, SD =12.10) also had significantly lower HEI scores compared to those with a college degree (M = 65.92, SD = 10.18). Additionally, individuals with monthly incomes of less than $1000 (M = 56.44, SD = 12.12) and between $1000 and $1999 (M = 57.25, SD = 11.55) had significantly lower scores compared to those with monthly income greater than $4000 (M = 64.69, SD = 10.52). Significant differences in diet quality scores were also found between the following sub-groups: homemakers (M = 70.25, SD = 13.34) compared to those unemployed (M = 55.64, SD = 11.25); those who received SNAP benefits (M = 55.18, SD = 9.59) compared to those who did not utilize any federal food assistance (M = 62.55, SD = 11.61); current smokers (M = 54.27, SD = 11.57) and former smokers (M = 60.41, SD = 10.78) compared with individuals who had never smoked (M = 64.34, SD = 11.50). Individuals diagnosed with lung (M = 63.63, SD = 11.96) and head and neck (M = 56.88, SD = 2.17) cancers had a significantly lower diet quality compared to those diagnosed with breast (M = 63.74, SD = 3.89) and endometrial or cervical (M = 71.86, SD = 4.57) cancers.

A regression model was constructed and included the following characteristics: age, gender, marital status, education, employment, monthly income, paying bills late, using federal and private food assistance, and smoking status. The model showed that four predictors explained 31.2% of the variance in HEI scores (*R*^2^ = 0.31, F (21, 194) = 4.20, *p* < 0.001). Having less than a high school degree, having a high school degree or GED, being a homemaker, and having endometrial or cervical cancer significantly predicted the HEI (Table 4). Individuals who received less than a high school degree or those who received a high school degree or GED had significantly lower HEI scores (*β* = −4.03, *p* = 0.04; *β* = −7.77, *p* = 0.001, respectively) compared to those with college degrees. Additionally, those who were homemakers had significantly higher HEI scores (*β* = 7.95, *p* = 0.008) compared to those who worked 40 or more hours per week. Also, individuals with endometrial or uterine cancer had significantly higher HEI scores (*β* = 12.56, *p* = 0.002) than individuals with breast cancer.

## 4. Discussion

We found this cohort of cancer patients to have a better diet quality compared to the general US population [22]. In addition, we found that having a high school degree or GED or less was predictive of a poorer diet quality, while being a homemaker and having endometrial or cervical cancer predicted a higher diet quality in this population.

Unlike previous studies among non-cancer populations [24], this current study did not find age, gender, and income to predict diet quality in this cohort of cancer patients. The inconsistency in findings could be due to the fact that our study sample differed demographically from the general non-cancer population with respect to age, gender, and income. Compared to the general US population, a higher proportion of our study sample were female and older. In addition, about one-third of the sample had a monthly income greater than $4000. Our finding that the education level was a determinant of diet quality is consistent with previous research where individuals with higher educational attainment reported higher diet quality compared to those with lower levels of education [13,14]. For instance, Hiza et al. showed that Americans with less than a high school education had higher scores for saturated fat and sodium compared to those with higher education levels, contributing to their poorer diet quality [25]. Consistent with this, cancer patients in our study that had only a high school education consumed significantly more saturated fatty acids than those with higher education (*p* < 0.0005). One possible explanation for this trend is that education might be associated with general knowledge about nutrition and the ability to translate that knowledge into better eating habits.

The finding that homemakers had higher diet quality than individuals with full-time jobs is a new addition to the literature. This result is contrary to some previous literature which suggests that the more the time spent at home, the poorer an individual’s diet quality, because of increased access to food and decreased structure in one’s eating schedule [26]. This finding could be explained by the fact that homemakers have more time to prioritize planning their meals and preparing their meals than individuals who work at least 40 hours per week. Despite the presence of a disease condition, it is speculated that perhaps, due to their strain for time to plan, individuals with full-time jobs may still make food choices based on convenience rather than health. Convenience is one of the four most important food choice motives, often rated above health in order of importance [27]. Typically, convenient foods such as fast food or prepackaged meals are processed, energy-dense, and lack fruits, vegetables, and whole grains [28]. Therefore, a poorer diet quality would be expected for individuals who have limited free time and are choosing foods based on convenience, such as those with a full-time job. Contrary to prior research, we found that individuals with endometrial or cervical cancer had significantly higher diet quality scores [29]. Our findings could be attributed to a higher proportion (70%) of participants with endometrial cancer having college education or higher education compared to participants with other cancers (range: 8.6%–55.6%).

This study addresses an important gap in the cancer literature by determining the patient characteristics associated with poor dietary consumption; however, it is not without limitations. First, the generalizability of our findings is limited, as the study utilized a convenient sample recruited from one Midwestern state in the United States. Caution is therefore warranted in the generalization of the study results to other cancer cohorts. In addition, because of limited resources, participants’ demographic and clinical data were self-reported instead of being extracted from medical records. This could be a potential limitation to the accuracy of the data. Similarly, the study is limited by the unavailability of data on the types of treatment received by the participants, as this could affect the dietary intake. Finally, one of the three recruitment sites for this study was a treatment center specific to breast and cervical cancer treatment. As a result, the gender and cancer diagnosis of the participants was slightly skewed towards women and breast cancer.

## 5. Conclusions

This study confirms that, in general, individuals diagnosed with cancer have an improved diet quality compared to the general population. However, there is disparity in the level of improvement based on specific demographic and disease-related characteristics. Our findings will help oncology healthcare providers easily identify and target cancer patients who are at increased risk for consuming poor-quality diets with much needed food resource interventions. Larger studies exploring the causal relationships between diet quality and demographic characteristics among cancer patients are warranted.

## Figures and Tables

**Table 1:** Sociodemographic Factors and Disease-related Characteristics by Healthy Eating Index (HEI) Scores among Cancer Patients (*N* = 242).

Characteristic	*n* ^a^	%	HEI 2010 Score Mean (SD)
Gender **			
Male	79	32.78	58.59 (11.08)
Female	162	67.22	62.96 (11.68)
Age *			
≤39 years	23	9.62	65.75 (12.26)
40–59 years	109	45.61	59.72 (12.43)
≥60 years	107	44.77	62.64 (10.38)
Marital Status **			
Married	145	60.17	63.63 (11.96)
Single/Never Married	26	10.79	58.53 (9.43)
Divorced/Widowed	65	26.97	58.13 (11.04)
Other	5	2.07	60.48 (9.22)
Education ***			
College Degree	89	36.93	65.92 (10.18)
1–2 Years College	53	21.99	60.97 (12.66)
HS Degree or GED	32	13.28	56.43 (12.10)
<HS Degree	67	27.80	58.58 (10.63)
Employment **^b^			
≥40 Hours/Week	79	32.78	61.72 (10.78)
<40 Hours/Week	34	14.11	61.75 (10.40)
Homemaker	15	6.22	70.25 (13.34)
Retired	80	33.20	62.05 (11.85)
Unemployed	33	13.69	55.64 (11.25)
Monthly Income ***			
≥$4000	82	35.04	64.69 (10.52)
$3000–$3999	40	17.10	63.61 (12.66)
$2000–$2999	48	20.51	60.63 (11.01)
$1000–$1999	41	17.52	57.25 (11.55)
<$1000	23	9.83	56.44 (12.12)
Pay Bills Late ***			
Yes	59	24.58	57.31 (10.69)
No	181	75.42	63.09 (11.68)
Federal Food Assistance **			
SNAP	28	11.62	55.18 (9.59)
Other	4	1.66	55.73 (16.39)
None	209	86.72	62.55 (11.61)
Private Food Assistance ***			
Yes	25	10.42	54.39 (10.40)
No	215	89.58	62.39 (11.53)
Smoking Status ***			
Current	30	12.40	54.27 (11.57)
Former	92	38.02	60.41 (10.78)
Never	120	49.59	64.34 (11.50)
Alcohol Use			
Yes	97	40.08	62.73 (11.04)
No	145	59.92	60.83 (12.05)

* *p* ≤ 0.05, ** *p* ≤ 0.01, *** *p* ≤ 0.001; ^a^ Because of missing data for some sociodemographic characteristics, not all categories add up to *N* = 242; *n*: subgroup sample size for each characteristic ^b^ Homemaker was differentiated from unemployed because homemakers are usually unemployed by choice. GED: General Education Diploma; HS: High school; SNAP: Supplemental Nutrition Assistance Program.

**Table 2:** Disease Characteristics by Healthy Eating Index (HEI) Scores among Cancer Patients.

Characteristic	*n* ^a^	% of *n*	HEI 2010 Score Mean (SD)
Type of Cancer ***			
Breast	88	38.43	63.74 (11.68)
Head/Neck	35	15.28	56.88 (9.06)
Ovarian	14	6.11	65.65 (8.85)
Lung	13	5.68	53.48 (9.50)
Skin	13	5.68	66.13 (9.60)
Endometrial	10	4.37	71.86 (9.95)
Rectal	11	4.80	55.22 (11.56)
Other Cancers	45	19.65	62.09 (11.63)
Time Since Diagnosis			
0–6 months	156	64.73	60.98 (11.81)
7 months to <2 years	38	15.77	63.35 (11.74)
2 year to <5 years	32	13.28	61.79 (12.05)
≥5 years	15	6.22	61.76 (8.54)
Stage of Cancer			
I	21	8.86	65.70 (13.26)
II	46	19.41	62.86 (9.91)
III	36	15.19	61.17 (11.48)
IV	71	29.96	59.39 (10.87)
Unknown	63	26.58	62.61 (12.94)

*** *p* ≤ 0.001; ^a^ Because of missing data for certain disease-related characteristics, not all categories add up to *N* = 242; *n*: subgroup sample size for each characteristic.

**Table 3:** Healthy Eating Index (HEI) Component Scores among Cancer Patients Compared to the National Population in 2009–2010.

Component	National Averages	Sample Averages	*p*-Value
Total HEI-2010	49.60	61.59 (11.67)	<0.001
Adequacy Components ^a^			
Total Vegetables	3.0	3.35 (1.26)	<0.001
Greens and Beans	1.2	2.78 (1.69)	<0.001
Total Fruit	2.4	2.99 (1.63)	<0.001
Whole Fruit	2.3	3.49 (1.55)	<0.001
Whole Grains	2.4	3.83 (2.59)	<0.001
Dairy	5.0	6.82 (2.50)	<0.001
Total Protein Foods	4.2	4.58 (0.74)	<0.001
Seafood & Plant Proteins	2.0	3.99 (1.30)	<0.001
Fatty Acids	5.0	4.70 (2.60)	0.077
Moderation Components ^b^			
Sodium	4.0	3.67 (2.65)	0.055
Refined Grains	5.9	8.87 (1.64)	<0.001
Empty Calories ^c^	12.1	12.51 (4.56)	0.160

^a^ These are to be consumed in adequate amounts; for these, a higher score indicates higher consumption. ^b^ These are to be consumed in moderate amounts; for these, a higher score indicates lower consumption; ^c^ Empty calories from solid fats, alcohol (intake above 13 g/100,000 cal), and added sugars.

**Table 4:** Socio-Demographic and Disease-Related Characteristics Predictive of Diet Quality.

	B	SE	*β*	*p*-Value	95% CI	
(Constant) ^a^	67.29	3.37		0.000	60.62	73.97
Female	−0.42	2.10	−0.02	0.84	−4.57	3.72
40–59 years	−5.04	2.59	−0.22	0.06	−10.15	0.07
≥60 years	−3.36	2.67	−0.15	0.21	−8.64	1.91
Single/Never Married	−3.23	2.41	−0.09	0.18	−7.98	1.52
<HS Degree	−4.03	1.90	−0.16	0.04	−7.78	−0.27
HS Degree or GED	−7.77	2.34	−0.23	0.001	−12.39	−3.14
Some College	−1.83	2.01	−0.07	0.36	−5.79	2.13
<40 hours/week	−3.35	2.11	−0.11	0.12	−7.52	0.82
Unemployed Homemaker ^b^	7.95	2.97	0.17	0.008	2.11	13.80
Unemployed Seeking Work	−2.34	2.26	−0.07	0.30	−6.79	2.11
$3000–$3999/month	0.79	1.90	0.26	0.68	−2.97	4.55
Pay Bills Late	−3.18	1.73	−0.12	0.07	−6.61	0.24
Other Fed Food Assistance	8.10	6.23	0.83	0.20	−4.28	20.47
Private Food Assistance	−2.05	2.83	−0.53	0.47	−7.64	3.54
Current Smoker	−3.93	2.36	−0.11	0.09	−8.59	0.73
Breast Cancer	4.36	2.44	0.19	0.08	−0.45	9.17
Ovarian Cancer	4.26	3.53	0.09	0.23	−2.71	11.23
Lung Cancer	−1.31	3.47	−0.03	0.71	−8.17	5.34
Skin Cancer	5.03	3.33	0.10	0.13	−1.54	11.61
Endometrial/Cervical Cancer	12.56	3.98	0.23	0.002	4.71	20.41
Other Cancers	3.08	2.36	0.11	0.19	−1.58	7.75

HS: High school; GED: General Education Diploma. ^a^ Reference categories include: male, ≤39 years, married, ≥college degree, unemployed, ≥$4000/month, do not pay bills late, no federal food assistance, no private food assistance, never smoker, and head/neck cancer; ^b^ Homemaker was differentiated from unemployed because homemakers are usually unemployed by choice.
